# Artificial Intelligence-Driven Biological Age Prediction Model Using Comprehensive Health Checkup Data: Development and Validation Study

**DOI:** 10.2196/64473

**Published:** 2025-04-11

**Authors:** Chang-Uk Jeong, Jacob S Leiby, Dokyoon Kim, Eun Kyung Choe

**Affiliations:** 1 Department of Biostatistics, Epidemiology & Informatics, Perelman School of Medicine, University of Pennsylvania, Philadelphia, PA, United States; 2 Institute for Biomedical Informatics, University of Pennsylvania, Philadelphia, PA, USA; 3 Department of Surgery, Seoul National University Hospital Healthcare System Gangnam Center, 38-40FL Gangnam Finance Center(prior Star Tower) 152, Teheran-ro, Gangnam-gu, Seoul, 06236, Republic of Korea, 82 221125500; 4 Department of Surgery, College of Medicine, Seoul National University, Seoul, 03087, South Korea

**Keywords:** biological age, aging clock, mortality, artificial intelligence, machine learning, record, history, health checkup, clinical relevance, gerontology, geriatric, older, elderly, aging, prediction, predictive, life expectancy, AI

## Abstract

**Background:**

The global increase in life expectancy has not shown a similar rise in healthy life expectancy. Accurate assessment of biological aging is crucial for mitigating diseases and socioeconomic burdens associated with aging. Current biological age prediction models are limited by their reliance on conventional statistical methods and constrained clinical information.

**Objective:**

This study aimed to develop and validate an aging clock model using artificial intelligence, based on comprehensive health check-up data, to predict biological age and assess its clinical relevance.

**Methods:**

We used data from Koreans who underwent health checkups at the Seoul National University Hospital Gangnam Center as well as from the Korean Genome and Epidemiology Study. Our model incorporated 27 clinical factors and employed machine learning algorithms, including linear regression, least absolute shrinkage and selection operator, ridge regression, elastic net, random forest, support vector machine, gradient boosting, and K-nearest neighbors. Model performance was evaluated using adjusted R^2^ and the mean squared error (MSE) values. Shapley Additive exPlanation (SHAP) analysis was conducted to interpret the model’s predictions.

**Results:**

The Gradient Boosting model achieved the best performance with a mean (SE) MSE of 4.219 (0.14) and a mean (SE) R^2^ of 0.967 (0.001). SHAP analysis identified significant predictors of biological age, including kidney function markers, gender, glycated hemoglobin level, liver function markers, and anthropometric measurements. After adjusting for the chronological age, the predicted biological age showed strong associations with multiple clinical factors, such as metabolic status, body compositions, fatty liver, smoking status, and pulmonary function.

**Conclusions:**

Our aging clock model demonstrates a high predictive accuracy and clinical relevance, offering a valuable tool for personalized health monitoring and intervention. The model’s applicability in routine health checkups could enhance health management and promote regular health evaluations.

## Introduction

Over the past several decades, global life expectancy has increased remarkably, rising from 66.8 years in 2000 to 73.4 years in 2019, according to the World Health Organization. However, healthy life expectancy has not kept pace, increasing only from 58.3 years to 63.7 years during the same period [1]. This demographic shift toward an aging population has led to increased health care dependency and associated social costs. The medical industry related to aging and the social costs thereof are continuously increasing [2]. Accurately assessing biological aging is a critical first step in mitigating age-related diseases and their socioeconomic impact.

Biological age refers to an estimation of an individual’s physiological and functional status, reflecting the cumulative effects of genetic, environmental, and lifestyle factors on the aging process [3]. An individual with a biological age younger than their chronological age may have a lower risk of developing age-related diseases, while an older biological age could indicate a higher vulnerability to such conditions. This highlights the clinical significance of accurately estimating the biological age for personalized health interventions and monitoring. While numerous studies have explored the human lifespan [4,5], their evaluation is challenging due to the required long-term observations and limited clinical applicability.

Applying findings from academic studies to clinical practice remains challenging. Current biological age prediction models, primarily based on conventional statistical methods such as multivariate regression analysis, rely on limited clinical data, restricting their predictive power and insights into the aging process [5-8]. Recent advances have led to models using omics data [9], including DNA methylation [10], transcriptome [11], metabolome [12], and telomere data [9]. However, these models face implementation challenges in clinical settings due to their complexity and the difficulty in measuring omics markers. The model requires multiple molecular modalities and functional data to exhibit a superior performance [9]. In addition, it is challenging to quantify the dynamic effects of environmental, lifestyle, behavioral, and interventional factors on biological age.

In Asian countries, the health checkup industry has been growing substantially [13-15], with individuals regularly monitoring their health status. However, these checkups typically only indicate normal or abnormal conditions for individual tests, lacking comprehensive health status indicators. Providing biological age predictions through an aging clock could serve as a valuable tool for the health screening of patients, offering a comprehensive health status measure and encouraging regular checkup participation.

This study investigated the clinical relevance of artificial intelligence (AI)-predicted biological age in the Korean population using comprehensive health checkup data, examining its relationship with various clinical characteristics.

## Methods

### Participants and Datasets

The study investigated the healthy population participating in comprehensive health checkups at the Seoul National University Hospital Gangnam center, from 2003 to 2016. The initial baseline data were used, and the participants included a total of 81,211 Koreans, who comprised the Health and Prevention Enhancement (H-PEACE) cohort. The details of the H-PEACE cohort have been described previously [16]. To summarize, each participant completed a questionnaire on their past medical history and underwent anthropometric measurements and laboratory tests after at least 10 hours of fasting on the same day. We also used the data from the Korean Genome and Epidemiology Study (KoGES) from the Korean Center for Disease Control and Prevention as the replication set. From the KoGES data, we used the health examination cohort (KoGES HEXA data), which included past medical history, anthropometric measurement, and laboratory data [17]. Briefly, the KoGES HEXA cohort is a national health examinee registry, consisting of 173,357 urban Korean adults who underwent health checkup programs. We used clinical factors overlapping between factors from the H-PEACE cohort data and the KoGES HEXA data. The exclusion criteria to define the healthy adult super-control cohort were as follows: (1) participants diagnosed with diabetes, hypertension, or dyslipidemia; (2) participants drinking alcohol more than 14 g/week; (3) current or previous smokers; (4) those aged less than 30 years; and (5) those having a history of malignant disease. To check the clinical implication of the predicted biological age, we performed multiclinical feature association study in the gene-environmental interaction and phenotype (GENIE) study [16], which consisted of 123 clinical factors and gene datasets. We used the 116 clinical factors, excluding the 27 factors used to predict biological age, to determine the multiple associations with predicted biological age.

### Development of a Biological Age Prediction Model

This study aimed to develop a widely applicable biological age model using only basic health screening parameters. The clinical features used to construct the biological age model were based on routine clinical measurements, including standard demographic features, blood test results, and anthropometric measurements, rather than the findings from expensive specialized examinations. The selection of clinical factors was constrained by the requirement that they be present in both the training dataset (H-PEACE cohort) and the replication dataset (KoGES HEXA cohort). Through this feature selection process, we used 27 clinical factors as inputs for predicting the biological age, which are gender, anthropometric measurements (height, weight, BMI, and waist circumference), metabolic status (levels of fasting glucose, glycated hemoglobin [HbA1c], uric acid, total cholesterol, triglyceride, high-density lipoprotein cholesterol, and low-density lipoprotein cholesterol), liver functions (albumin, total bilirubin, alkaline phosphatase, aspartate aminotransferase, alanine aminotransferase, and gamma-glutamyl transferase), complete blood cell counts (white blood cell count, red blood cell count, hemoglobin, hematocrit, and platelet count), calcium and renal function (levels of blood urea nitrogen and creatinine, and the glomerular filtration rate calculated using the chronic kidney disease epidemiology collaboration (CKD-EPI) equation [18]).

The true labels were defined as the chronological age of the super-control population, based on the assumption that chronological age aligns with biological age in physiologically standard individuals. This cohort was carefully selected to exclude individuals with pathological conditions, such as metabolic diseases or malignant diseases, as well as those exposed to environmental factors known to influence biological age, including smoking and alcohol consumption [19].

The baseline data of the H-PEACE super-control cohort was split into 80% as the training set and 20% as the testing set with stratification based on both age and sex. Specifically, age stratification was conducted by categorizing participants into decade-based intervals, which were 20‐29, 30‐39, 40‐49, 50‐59, 60‐69, 70‐79, and more than 80 years. Five-fold cross-validation was performed to find the best hyperparameter for each model. We employed various machine learning models such as linear regression, least absolute shrinkage and selection operator (LASSO) regression, ridge regression, elastic net, random forest, support vector machine (SVM), gradient boosting, and K-nearest neighbors. These models were chosen for their ability to handle diverse relationships in data, including linear, nonlinear, and complex interactions. Hyperparameter optimization for each model was conducted using a grid search. To evaluate the performances of the developed models to predict biological age, we used adjusted R^2^ and mean squared error (MSE) values. The evaluation results reported in this study are from 10 iterative experiments. A replication study was conducted in the KoGES HEXA dataset with the same experimental setting as the H-PEACE dataset. We interpreted the biological age prediction results using Shapley Additive exPlanation (SHAP) [20]. SHAP analysis was performed to elucidate the roles and impacts of different biological markers in predicting biological age.

### Investigation of the Clinical Relevance of the Predicted Biological Age

We conducted linear regression analysis for multiple clinical factors using the GENIE study dataset with the predicted biological age, adjusting for chronological age. All reported *P* values were corrected for multiple tests using the Bonferroni correction.

**Figure 1.**
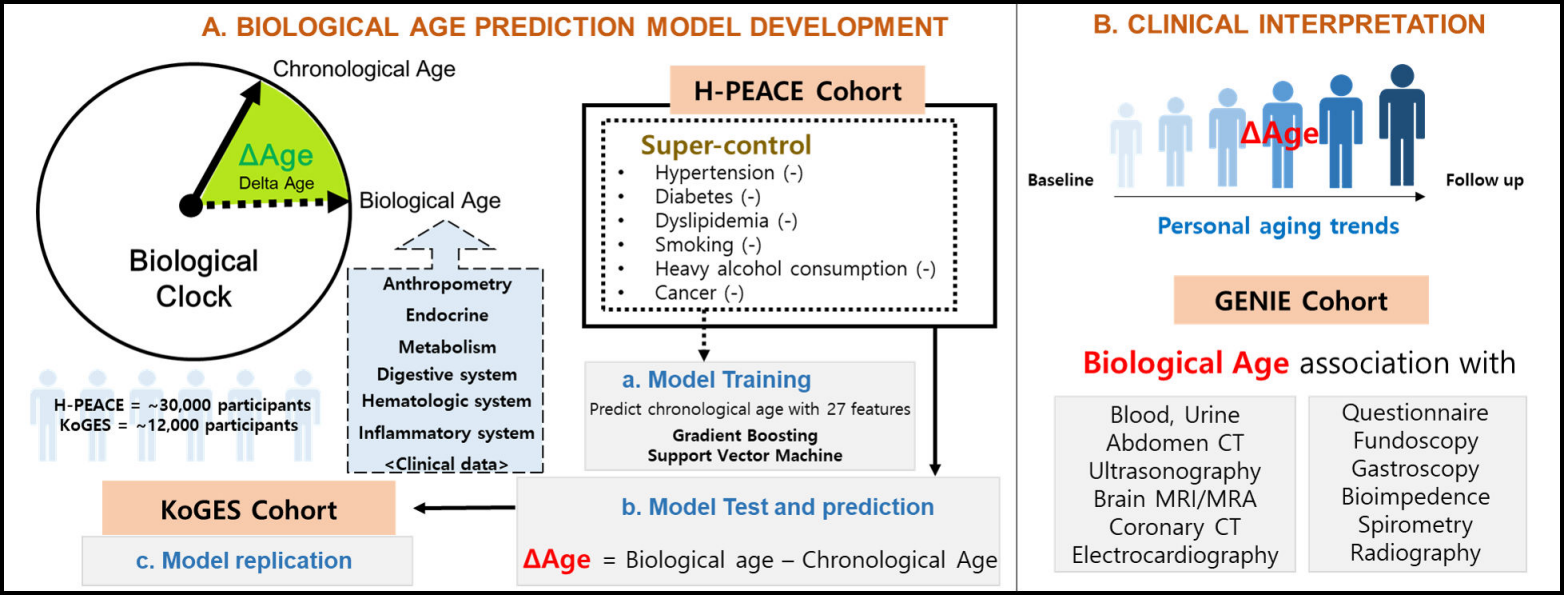
Overview of the study. H-PEACE: Health and Prevention Enhancement, KoGES: Korean Genome and Epidemiology Study, GENIE: gene-environmental interaction and phenotype, CT: computed tomography, MRI: magnetic resonance imaging, MRA: magnetic resonance angiography.

The overview of the study is shown in Figure 1.

### Ethical Considerations

The Institutional Review Board of the Seoul National University Hospital approved the study protocol and waived the need for informed consent (IRB number H-2005-223-1129). The study was performed in accordance with the Declaration of Helsinki. All patient data were deidentified before analysis, and strict confidentiality measures were implemented throughout the data collection, storage, and analysis processes. Access to the data was restricted to authorized research personnel only. The data management procedures complied with relevant data protection regulations to safeguard participants' privacy. No personally identifiable information was included in the final dataset or results.

### Statistical and Computational Analyses

All analyses and calculations were performed using Python version 3.11.11 (Python Software Foundation). Multiple evaluation indices, including adjusted R^2^ and MSE, β, and *P* value, were used to comprehensively evaluate the performances of the models and the significance of the associations. The statistical significance was based on a two-tailed *P* value of <.05.

## Results

### General Characteristics of the Participants

After applying the exclusion criteria to define the super-control cohort and removing the missing variables, we developed a model using a dataset collected from 28,417 individuals who underwent comprehensive health checkups at the Seoul National University Hospital Gangnam Center (ie, the H-PEACE cohort). The enrollment process is shown in Figure S1 in Multimedia Appendix 1. The mean (SD) participant age was 44.22 (11.26) years for 6467 men and 21,950 women. The baseline characteristics according to the aging of the study participants are shown in Table S1 in Multimedia Appendix 2. There were 1005 participants who were more than 65 years old and 27,412 who were 65 years old or less. Figure S2 in Multimedia Appendix 1 shows the chronological age distribution for the respective genders. Figure S3 in Multimedia Appendix 1 shows the distribution of gender in the training and test sets as well as the chronological age group distribution in the training and test sets.

### Development and Performance of the Biological Age Prediction Model

The models were trained using 27 clinical variables on the full training dataset (80% of the super-control cohort), then predicted on the test set (20%). For generalizability, 5-fold cross-validation with 10 iterative experiments were performed. The model was replicated using the KoGES HEXA data of 11,968 super-controls. Among 8 machine learning algorithms, the model showing the best performance was gradient boosting, for which the mean (SE) MSE value was 4.219 (0.140) and the mean (SE) R^2^ value was 0.967 (0.001). The hyperparameters for the gradient boosting model were α=0.9, complexity parameter α=0, learning rate=0.1, maximum depth=5, and number of trees=500.

The second-best performing algorithm was the SVM model, with a mean (SE) MSE of 8.244 (0.210) and mean (SE) R^2^ value of 0.935 (0.002). The performances of the 8 machine learning models in the test set are shown in Table 1.

**Table 1.** Comparison of the performances of 8 machine learning models to predict biological age in the test set (N=5684).

Model	Mean squared error, mean (standard error)	R^2^, mean (standard error)
K nearest neighbor	63.829 (0.176)	0.497 (0.002)
Elastic net	50.518 (2.251)	0.602 (0.018)
Linear regression	50.314 (5.165)	0.603 (0.040)
LASSO^a^	50.271 (3.477)	0.604 (0.027)
Ridge	50.235 (5.047)	0.604 (0.039)
Random forest	20.941 (1.020)	0.835 (0.008)
Support vector machine	8.244 (0.210)	0.935 (0.002)
Gradient boosting	4.219 (0.140)	0.967 (0.001)

a LASSO: Least Absolute Shrinkage and Selection Operator.

**Figure 2.**
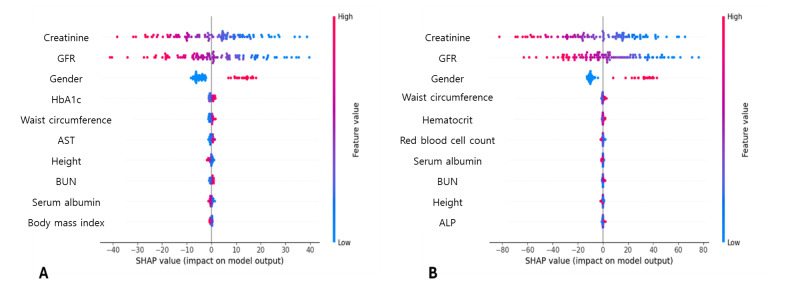
Visualization of the feature importance with Shapley Additive exPlanation (SHAP) values. SHAP summary plots for the model from the gradient boosting model (A) and the support vector machine (SVM) model (B) are visualized. Features with broader spreads and higher SHAP values have a more significant impact to predict biological age and values with the color gradient indicate whether higher or lower feature values are associated with increased biological age predictions. GFR: glomerular filtration rate, HbA1c: glycated hemoglobin, AST: aspartate aminotransferase, BUN: blood urea nitrogen, ALP: alkaline phosphatase.

The SHAP values from the gradient boosting and SVM models were analyzed to interpret their predictions of biological age. The SHAP values offer feature importance, providing the interpretation of the model’s decision-making by quantifying the contribution of each feature to the model’s output [20]. The corresponding visualizations are presented in Figure 2.

In the predictions of biological age generated by the gradient boosting model, the markers of kidney function, gender, HbA1c level, liver function, and anthropometric measurements were highlighted as significant predictors. In the SVM model, the SHAP summary plot revealed that kidney function markers, gender, liver function markers, red blood cell indices, and anthropometric measurements were the most influential predictors of biological age. These findings underscored the multifaceted nature of aging and highlighted the importance of maintaining optimal kidney function, metabolic status, and body composition in mitigating biological aging.

### Subgroup Analysis by Gender

The performances of the gradient boosting and SVM models were evaluated in the respective genders, male versus female participants. Applying the gradient boosting model to male participants, the mean (SE) MSE value was 5.258 (0.490). The SHAP value was significantly influenced by renal function, metabolic status, red blood cell indices, and anthropometric measurements. In female participants, the mean (SE) MSE value was 2.743 (0.099) and the SHAP values with the highest impact were similar to those for the male participants. The performances in the SVM and SHAP plots for male and female participants are shown in Table 2 and Figure S4 in Multimedia Appendix 1.

**Table 2.** Comparison of the performances of the test set and replication set in all ages and in age above 65 years.

	Gradient boosting		Support Vector Machine	
Dataset	Subgroup	All ages	Age above 65 years	All ages	Age above 65 years
		MSE^a^	MSE^a^	MSE^a^	MSE^a^
Test set	Male and Female	4.219 (0.140)	18.942 (1.250)	8.244 (0.210)	18.077 (1.094)
Replication set	Male and Female	2.406 (0.083)	2.190 (0.085)	7.838 (0.065)	3.116 (0.148)
Test set	Male	5.258 (0.490)	20.357 (2.493)	15.476 (0.652)	20.678 (1.504)
Replication set	Male	2.032 (0.110)	2.096 (0.113)	6.337 (0.053)	3.143 (0.205)
Test set	Female	2.743 (0.099)	17.153 (0.819)	7.955 (0.170)	18.928 (1.698)
Replication set	Female	16.050 (0.467)	3.746 (0.366)	37.301 (0.777)	3.832 (0.510)

a Mean squared error (MSE) values are shown as mean (standard error).

### Replication of the Developed Biological Age Prediction Model

We replicated the model with the gradient boosting and SVM models using the KoGES HEXA data for participants of all ages and those under 65 years of age. The prediction model was better replicated in male participants and those aged above 65 years. The replicated results are shown in Table 2.

### Delta Age Evaluation Using Baseline and Follow-Up Data

The model predicted the biological age in the test set (20% of the total dataset; 5684 individuals). The delta age, which is the difference between the biological age and the predicted biological age, was calculated for all individuals in the test dataset.

**Figure 3.**
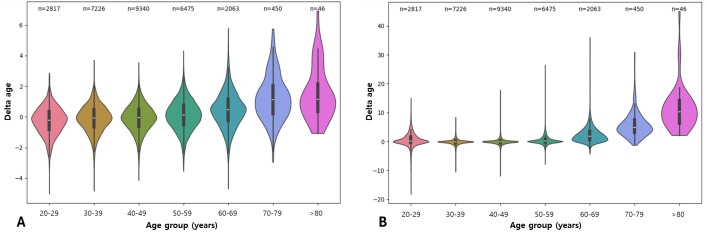
The delta age distribution across different age groups. The distribution of the delta age across different age groups in the test set at baseline (total n=5684) is shown for the gradient boosting (A) and support vector machine (B) models.

Figure 3 shows the delta age distribution across the different age groups, and Figure S5 in Multimedia Appendix 1 shows the delta age distribution across different age groups in each gender.

With the increase in the age groups, there was an observable pattern in the variability of delta age. In the younger age group (the 1st to the 3rd groups), there was increased variability, suggesting diverse aging processes influenced by multiple factors such as genetics, lifestyle, and health conditions. Older groups (the 7th and 8th groups) showed less variability and more positive values of delta age, possibly reflecting a selection of healthier individuals who have managed to reach older ages. The results were consistent in each gender group as well.

Among 5684 baseline participants, there were 2022 participants who had follow-up data, with follow-up duration ranging from 1 to 10 years (with 0 meaning the same age at two consecutive visits).

**Figure 4.**
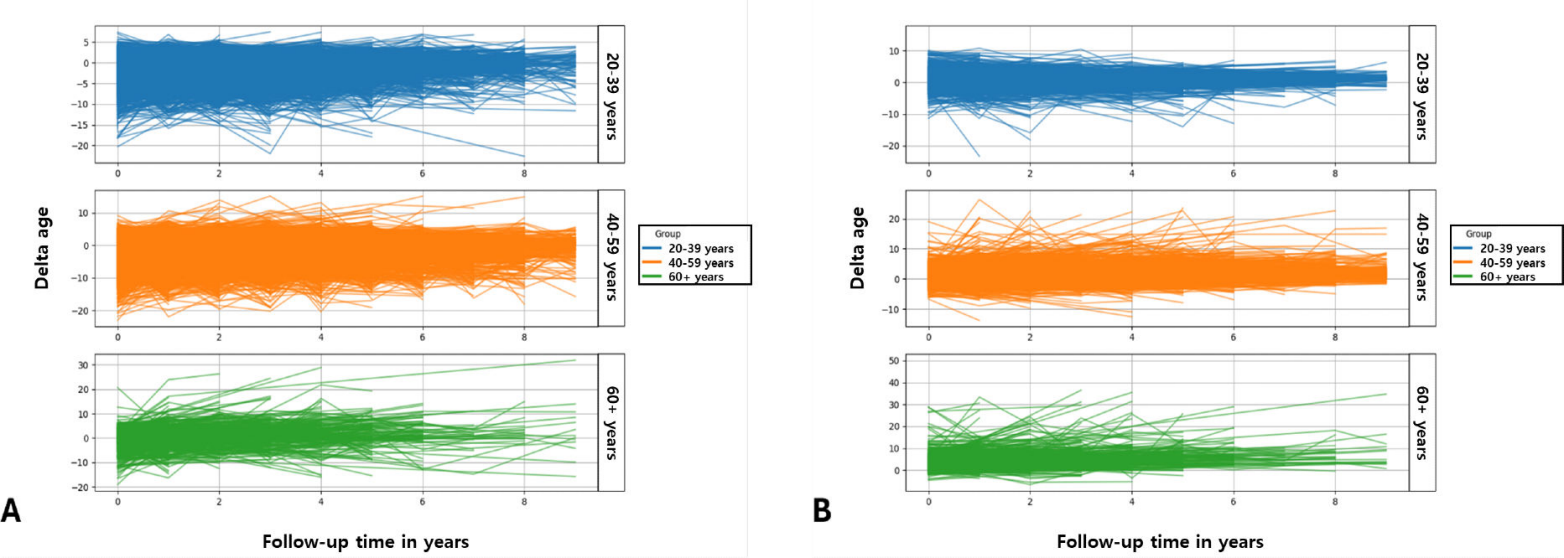
The delta age trajectory during follow-up across different age groups. The projectiles of the changes in delta age during follow-up across different age groups in the test set (n=2022) are shown for the gradient boosting model (A) and SVM model.

Figure 4 shows the projectiles of the changes in delta age during follow-up across different age groups in the test set (n=2022) for the gradient boosting and SVM models. While the younger age groups exhibited stable biological aging trajectories, the middle-aged and older groups showed increased variability and accelerated aging over the follow-up period. The observed trends were consistent across both the gradient boosting and SVM models, providing robust evidence for the described aging patterns.

Figure S6 in Multimedia Appendix 1 shows the trajectory of the changes in delta age during follow-up across different gender groups in the test set at baseline (n= 5684) for the gradient boosting model and SVM model.

### Clinical Interpretation Using a Multiple Clinical Factor Association Study

To explore the clinical relevance of the predicted biological age, we performed a phenome-wide association analysis between 116 phenotypes corroborated by comprehensive health checkups from the GENIE study dataset and the output of the predicted biological age model (data for both genders are shown in Table S2 in Multimedia Appendix 2, data for the male gender are shown in Table S3 in Multimedia Appendix 2, and data for the female gender are shown in Table S4 in Multimedia Appendix 2).

We found that the predicted biological age was significantly associated with 50 clinical factors after Bonferroni correction (Tables S2-S4 in Multimedia Appendix 2). Notably, the most significant associations were observed with metabolic status, body composition, fatty liver, smoking status, and pulmonary function, after adjusting for the chronological age. Table 3 shows the top 10 significant results in both genders, in male participants, and in female participants. The correlations among the variables are shown in Figure S7 in Multimedia Appendix 1. These findings suggest that the predicted biological age, developed from multiple clinical factors from comprehensive health checkups, may also possess predictive capabilities for aging in various organs.

**Table 3.** Top 10 significant clinical factors associated with the predicted biological age.

Clinical factors	Study modality	N	*β*	*P* value
In both genders				
Diabetes diagnosis	Questionnaire	10,351	−.002	1.75×10^−270^
Skeletal muscle mass	Bioelectrical impedance	10,231	−.053	7.87×10^−256^
Fatty liver	Ultrasonography	10,287	−.006	5.20×10^−195^
Diabetes medication	Questionnaire	10,351	−.001	4.38×10^−178^
Metabolic syndrome	Questionnaire	10,351	−.003	1.35×10^−174^
Smoking status	Questionnaire	8995	−.004	8.43×10^−170^
Visceral fat area	Abdominal computed tomography	6183	−46.952	2.86×10^−153^
Forced vital capacity (liters)	Spirometry	10,138	−.005	6.32×10^−141^
Forced expiratory volume in 1 s (liters)	Spirometry	10,138	−.004	5.71×10^−135^
Chloride level	Blood	9964	.011	4.18×10^−77^
In male participants				
Diabetes diagnosis	Questionnaire	4292	−.001	1.91×10^−56^
Fatty liver	Ultrasonography	4270	−.004	2.74×10^−46^
Metabolic syndrome	Questionnaire	4292	−.002	1.72×10^−41^
Diabetes medication	Questionnaire	4292	−.001	9.63×10^−36^
Mean corpuscular hemoglobin	Blood	4264	.013	3.61×10^−35^
Mean corpuscular volume	Blood	4264	.026	1.54×10^−27^
Visceral fat area	Abdominal computed tomography	2157	−26.868	3.61×10^−24^
Total fat area	Abdominal computed tomography	2157	−48.823	1.23×10^−14^
Mean corpuscular hemoglobin concentration	Blood	4264	.005	5.10×10^−14^
Heart rate	Electrocardiography	3297	−.040	1.02×10^−12^
In female participants				
Diabetes diagnosis	Questionnaire	6059	−.002	4.13×10^−146^
Diabetes medication	Questionnaire	6059	−.002	2.49×10^−98^
Metabolic syndrome	Questionnaire	6059	−.002	1.93×10^−38^
Fatty liver	Ultrasonography	6017	−.004	5.03×10^−31^
Potassium level	Blood	5839	.001	4.02×10^−24^
Sodium level	Blood	5839	.007	1.59×10^−23^
Chloride level	Blood	5839	.007	3.07×10^−15^
Forced vital capacity (liters)	Spirometry	5956	.002	3.97×10^−15^
Forced vital capacity percent	Spirometry	5956	.032	4.71×10^−14^
Visceral fat area	Abdominal computed tomography	4026	−17.835	9.80×10^−14^

## Discussion

This study underscores the clinical relevance of biological age as predicted by AI using comprehensive health checkup data. Our findings elucidate the clinical relevance of biological age—as assessed through machine learning models—which exhibits strong associations with multiple clinical factors, thereby providing valuable insights into the aging process and its implications on health. The result demonstrated that the best-performing model, gradient boosting, achieved a mean (SE) MSE value of 4.219 (0.140) and mean (SE) R^2^ value of 0.967 (0.001), indicating acceptable predictive accuracy. Additionally, the SHAP analysis highlighted markers such as kidney function, gender, HbA1c level, liver function, and anthropometric measurements as significant predictors of biological age. These findings suggest that biological age is a multifaceted construct influenced by various physiological factors and can serve as a robust indicator of the overall health status. The identification of these markers is significant because it supports the importance of maintaining optimal kidney function, metabolic health, and body composition in mitigating biological aging. This comprehensive understanding can lead to more targeted and effective interventions aimed at improving overall health and longevity.

Our study findings align with existing research findings that emphasize the utility of biological age as a comprehensive health indicator [21-23]. Previous studies have also identified the importance of factors such as kidney function [24], metabolic health [25,26], and inflammatory markers [27,28] in the aging process. However, our use of machine learning models such as the gradient boosting model and the SVM model provides a more nuanced understanding of how these factors interact to influence biological age, setting our research apart from traditional statistical methods. For instance, while existing literature has demonstrated the importance of individual biomarkers, our approach integrates multiple clinical variables to provide a holistic prediction model.

The use of multiple machine learning algorithms allowed for a comprehensive evaluation of their predictive capabilities. The gradient boosting model outperformed other algorithms, with performance R² of 0.967 and MSE of 4.219, due to its ability to handle nonlinear relationships and feature interactions, which are crucial in modeling complex biological systems [29]. Unlike linear models such as the ridge or LASSO regression, the gradient boosting model iteratively optimizes residual errors, capturing intricate dependencies between features. Its robustness to outliers and noise further enhances performance, making it particularly suited for real-world health checkups [30,31]. The generalizability of gradient boosting, demonstrated in both the training and replication datasets, underscores its potential for clinical application. Future studies should explore hybrid approaches to integrate the strengths of the gradient boosting model with more efficient models for broader use.

In the SHAP analysis, kidney function emerged as a significant predictor of biological age, consistent with its established role as an indicator of systemic health [32]. The decrease in kidney function is closely associated with aging-related changes, such as a reduced glomerular filtration rate and increased risk of chronic kidney disease [33]. These conditions often signify cumulative damage from metabolic stressors, hypertension, and other age-related factors [34]. The inclusion of kidney function markers, such as creatinine and the estimated glomerular filtration rate, in our model highlights their critical contribution to capturing the physiological aging process. Maintaining optimal kidney function could therefore serve as a target for mitigating biological aging and reducing the burden of associated comorbidities.

The body composition, including markers such as waist circumference and BMI, was another key contributor identified on SHAP analysis. This finding underscores the impact of adiposity and muscle mass on aging trajectories. Increased visceral fat and reduced skeletal muscle mass are hallmark features of sarcopenic obesity, a condition linked to metabolic dysfunction and accelerated biological aging [35,36]. Such changes exacerbate systemic inflammation and insulin resistance, further compounding aging-related risks [37,38]. The identification of body composition as a significant predictor emphasizes the importance of lifestyle interventions, such as exercise and dietary modifications, to preserve muscle mass and manage body fat. By integrating these markers into the prediction model, our study highlights their multifaceted roles in biological aging. Kidney function and body composition serve not only as indicators of systemic health but also as modifiable factors, offering potential avenues for personalized health interventions aimed at slowing biological aging and promoting longevity. This enhanced understanding enriches the clinical utility of our model and provides actionable insights for practitioners.

Visualization of the delta age distribution across different age groups shows that there is increased variability in the younger age group and less variability and a more positive value of delta age in the older group. This pattern underscores the importance of middle age as a critical period for implementing health interventions to manage biological aging effectively, and the need for focused care strategies to manage aging-related health issues in older adults.

In the multiple clinical factor association study for predicted biological age, we found that the predicted biological age was significantly associated with 50 clinical factors even after the adjustment of chronological age. The most significant associations were observed with the metabolic status, body composition, fatty liver, smoking status, and pulmonary function. These associations highlight the clinical relevance of biological age as a comprehensive marker of an individual’s health status and suggest potential pathophysiological mechanisms accompanying the aging process. Reduced pulmonary function is known to be an indicator of systemic aging and has been linked to increased mortality and morbidity in older adults [39,40]. This suggests that maintaining optimal lung function could be crucial in mitigating the effects of biological aging and improving overall health outcomes.

The association between smoking status and predicted biological age is well-documented in various studies. For example, research using AI to analyze blood data from 149,000 adults revealed that smokers exhibit a faster rate of biological aging compared to nonsmokers [41]. Remarkably, female smokers were predicted to have a biological age twice that of their chronological age [42]. These findings emphasize the detrimental impact of smoking on biological age. Additionally, exposure to tobacco during early life has been linked to accelerated biological aging in adulthood [43]. The underlying mechanisms include inflammation [44,45], epigenetic changes [46], and chromosomal damage [47]. These results underline the potential for biological recovery after cessation, aligning with prior research and emphasizing both the dangers of smoking and the benefits of quitting.

Body composition changes, including increased body fat, decreased muscle mass, and higher BMI, were also significantly associated with biological age. These findings align with previous studies that have shown that sarcopenia (loss of muscle mass) and obesity are critical factors in the aging process [48]. Excess body fat, particularly visceral fat, is associated with metabolic syndrome, cardiovascular diseases, and insulin resistance, all of which contribute to accelerated biological aging [49,50]. Conversely, maintaining muscle mass is essential for physical function and metabolic health, highlighting the importance of exercise and nutrition in managing the biological age. These findings also provide insights into the pathophysiological aspects of aging, suggesting that targeted interventions in these areas could potentially mitigate the adverse effects of aging and improve health outcomes.

The results have significant implications for health care and aging research. By providing a comprehensive measure of biological age, health care providers can better assess an individual’s overall health status and identify potential risks for age-related diseases. This approach could also facilitate more personalized health care strategies, improving patient outcomes and quality of life. Furthermore, the findings contribute to the broader understanding of the aging process, offering valuable insights for researchers aiming to develop innovative strategies to overcome age-related diseases and enhance healthy aging.

Despite the robust findings, there are limitations to consider. The study population was predominantly Korean, which may limit the generalizability of the results to other ethnic groups. Additionally, the reliance on clinical data from health checkups may not capture all factors influencing biological age, such as genetic predispositions or epigenetic clocks and environmental influences. Future studies should aim to include more diverse populations and incorporate additional variables to enhance the comprehensiveness and applicability of the models. Furthermore, longitudinal studies are needed to validate the long-term predictive capabilities of the models and their effectiveness in different health care settings.

In conclusion, our study demonstrates the clinical relevance of biological age as predicted by machine learning models. The findings provide valuable insights into the aging process and highlight the potential of biological age as a comprehensive health indicator. Future research should focus on refining these models and exploring their applicability in diverse populations to further enhance their utility in clinical practice. The integration of advanced machine learning techniques with comprehensive health data holds great promise for advancing our understanding of aging and improving health care outcomes.

## Supplementary material

10.2196/64473 Multimedia Appendix 1 Supplementary figures.

10.2196/64473 Multimedia Appendix 2 Supplementary tables.

## Abbreviations

AI: artificial intelligence
CKD-EPI: chronic kidney disease epidemiology collaboration
GENIE: gene-environmental interaction and phenotype
H-PEACE: Health and Prevention Enhancement
HbA1c: glycated hemoglobin
HEXA: health examination
KoGES: Korean Genome and Epidemiology Study
LASSO: least absolute shrinkage and selection operator
MSE: mean squared error
SHAP: Shapley Additive exPlanation
SVM: support vector machine
