# Supplementary material for: Artificial Intelligence-Driven Biological Age Prediction Model Using Comprehensive Health Checkup Data: Development and Validation Study
Source: JMIR Aging. 2025 Apr 11;8:e64473. doi: 10.2196/64473 (PMC12007724; doi:10.2196/64473)
Supplement: Multimedia Appendix 1 [file aging-v8-e64473-s001.pdf]

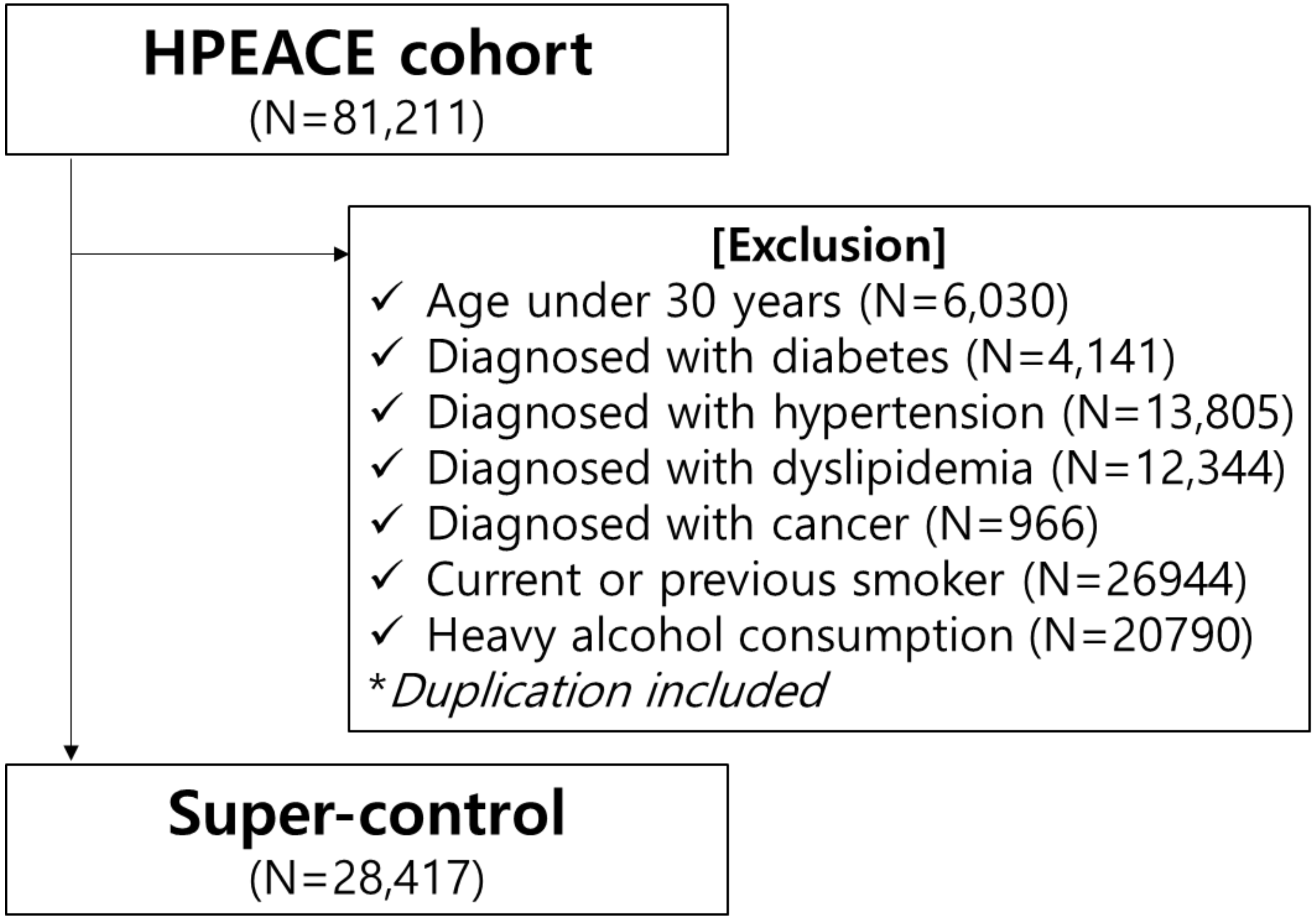

Figure S1. Enrollment process of the super-control in H-PEACE cohort

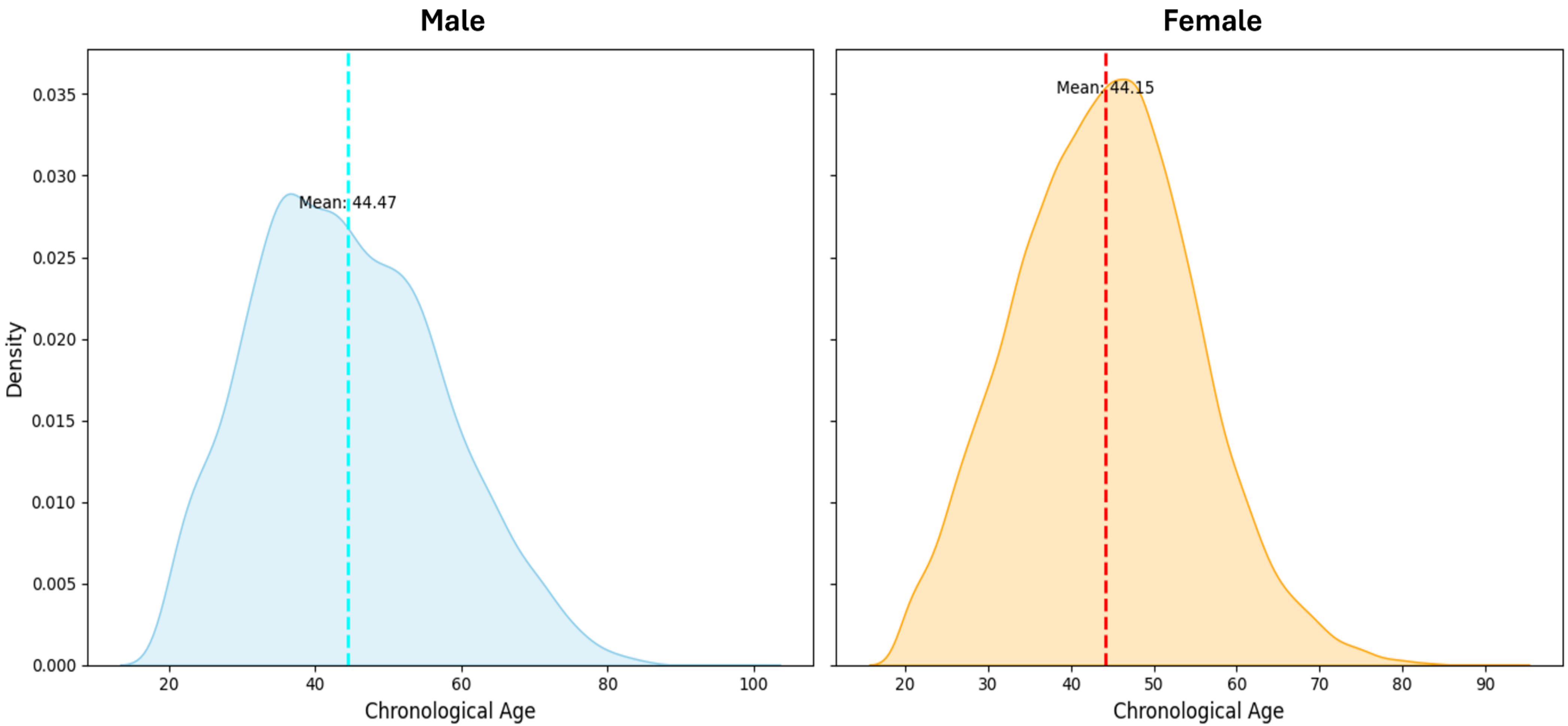

Figure S2. Distribution of the chronological age in H-PEACE cohort (total super-control group) in respective gender.

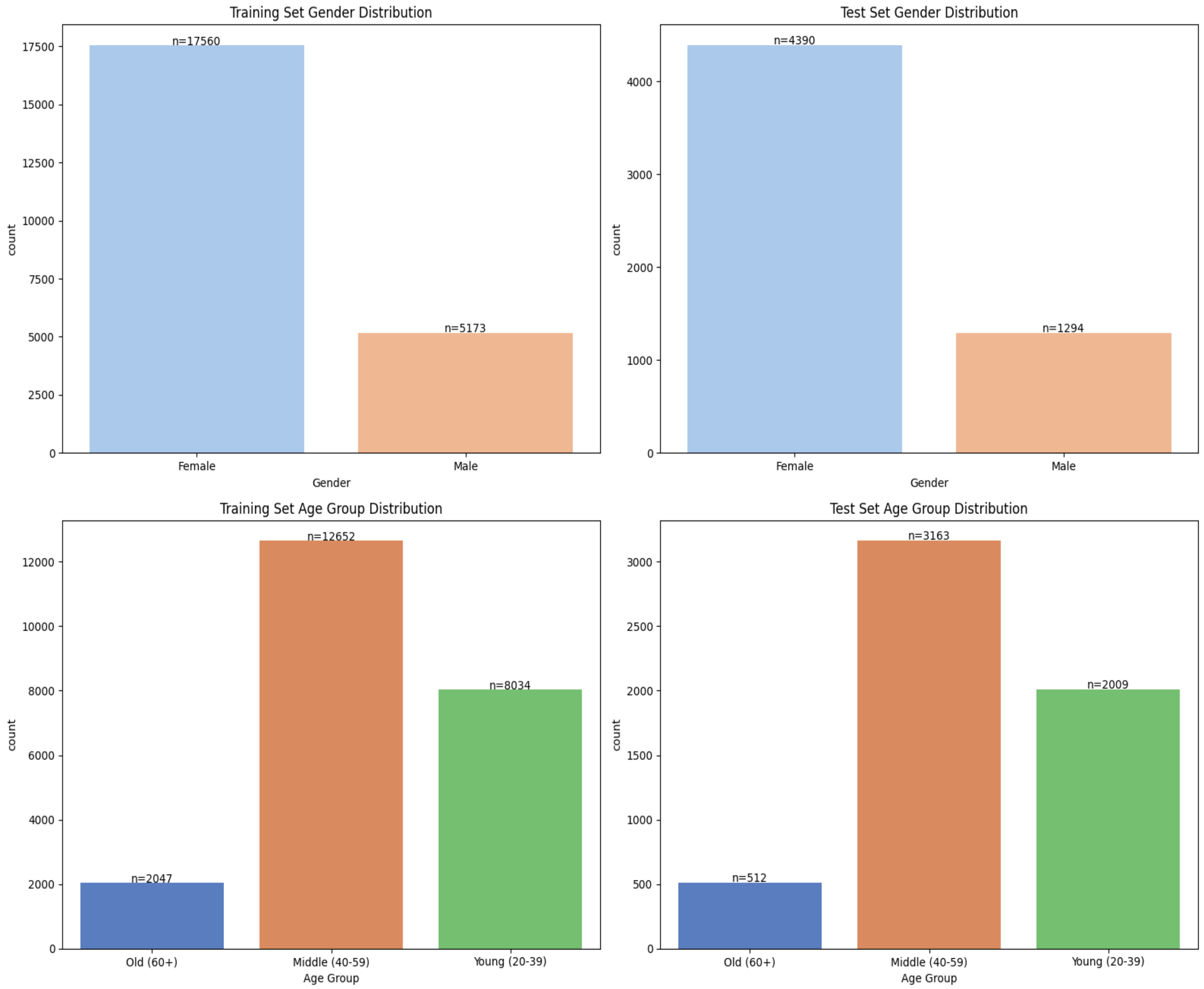

Figure S3. Distribution of the gender and chronological age group in H-PEACE cohort in train and test set, respective gender.

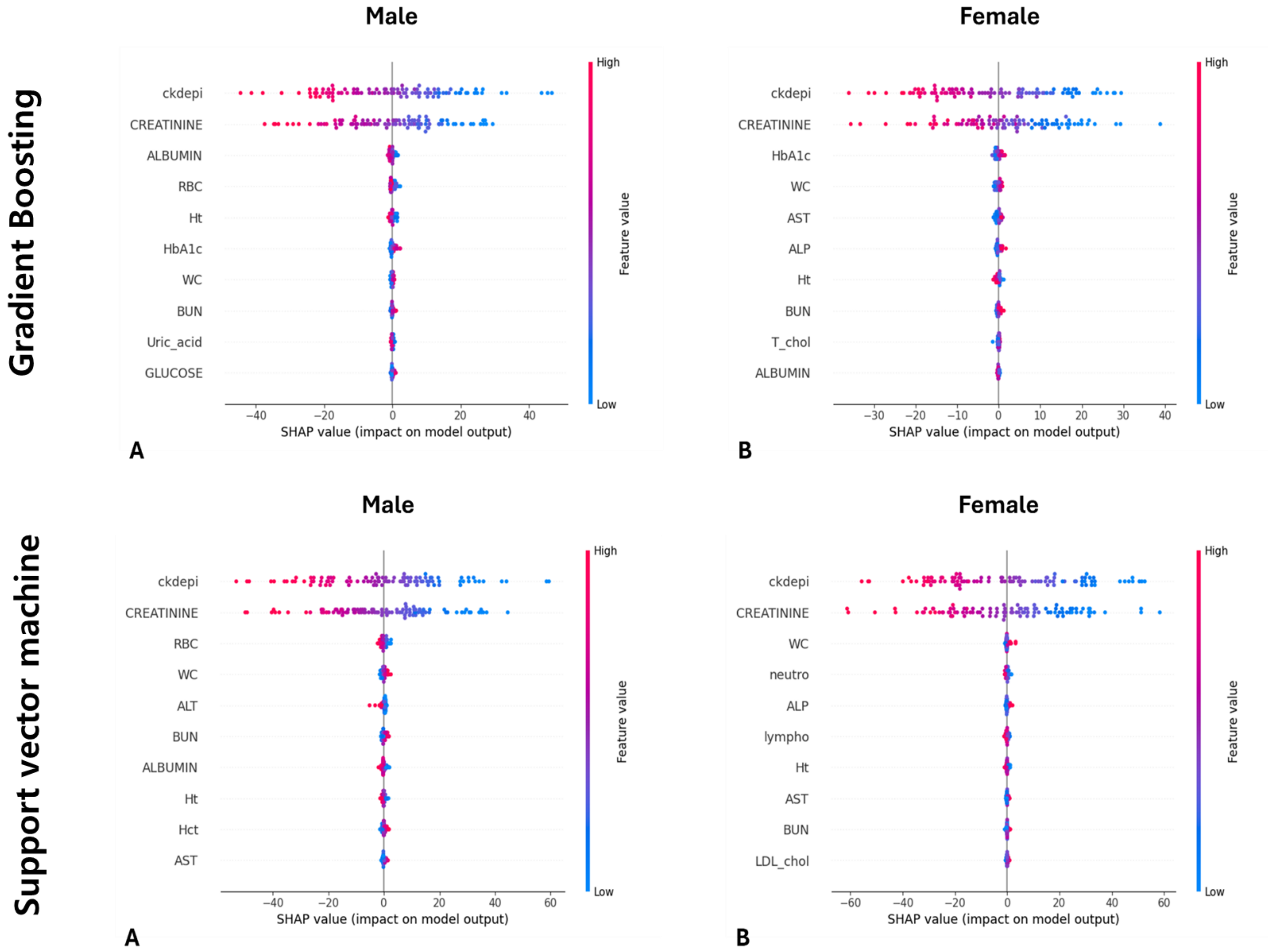

Figure S4. Visualization of the feature importance with SHAP value in male and female, respectively

Gradient Boosting

Support Vector Machine

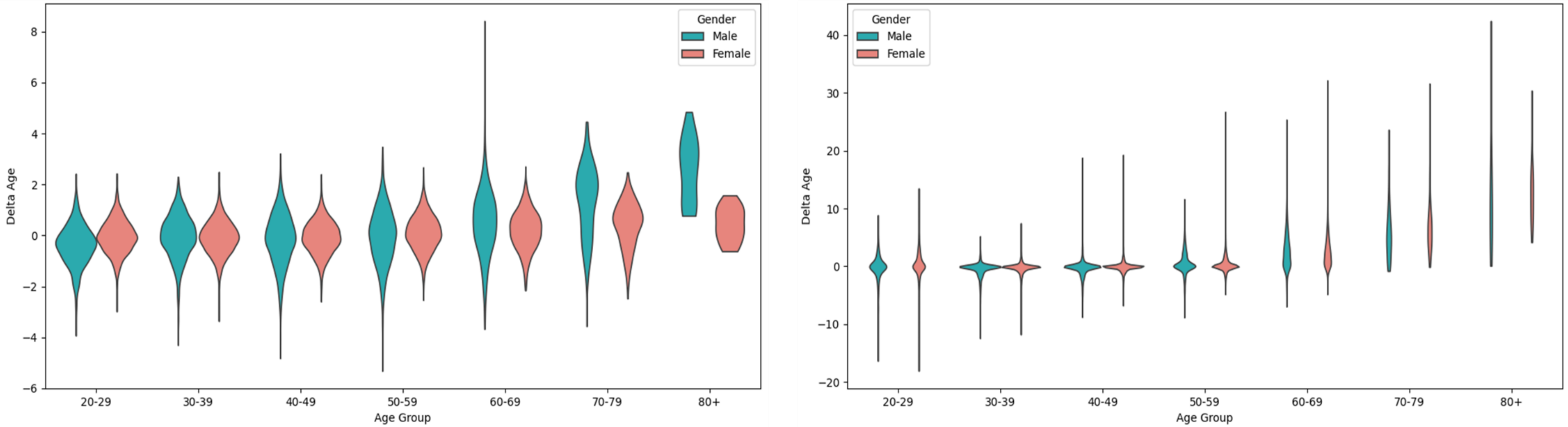

A

B

Figure S5. The delta age distribution across different age group in each gender

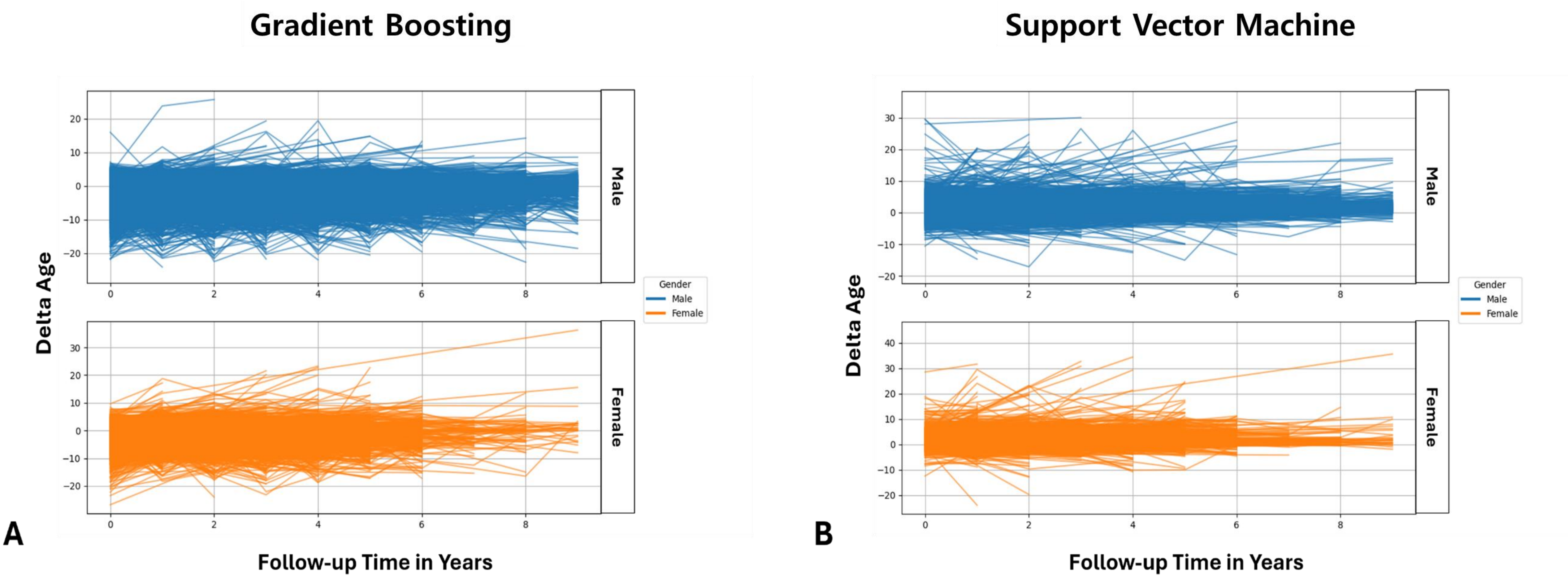

Figure S6. The delta age trajectory during follow up across different age group in each gender

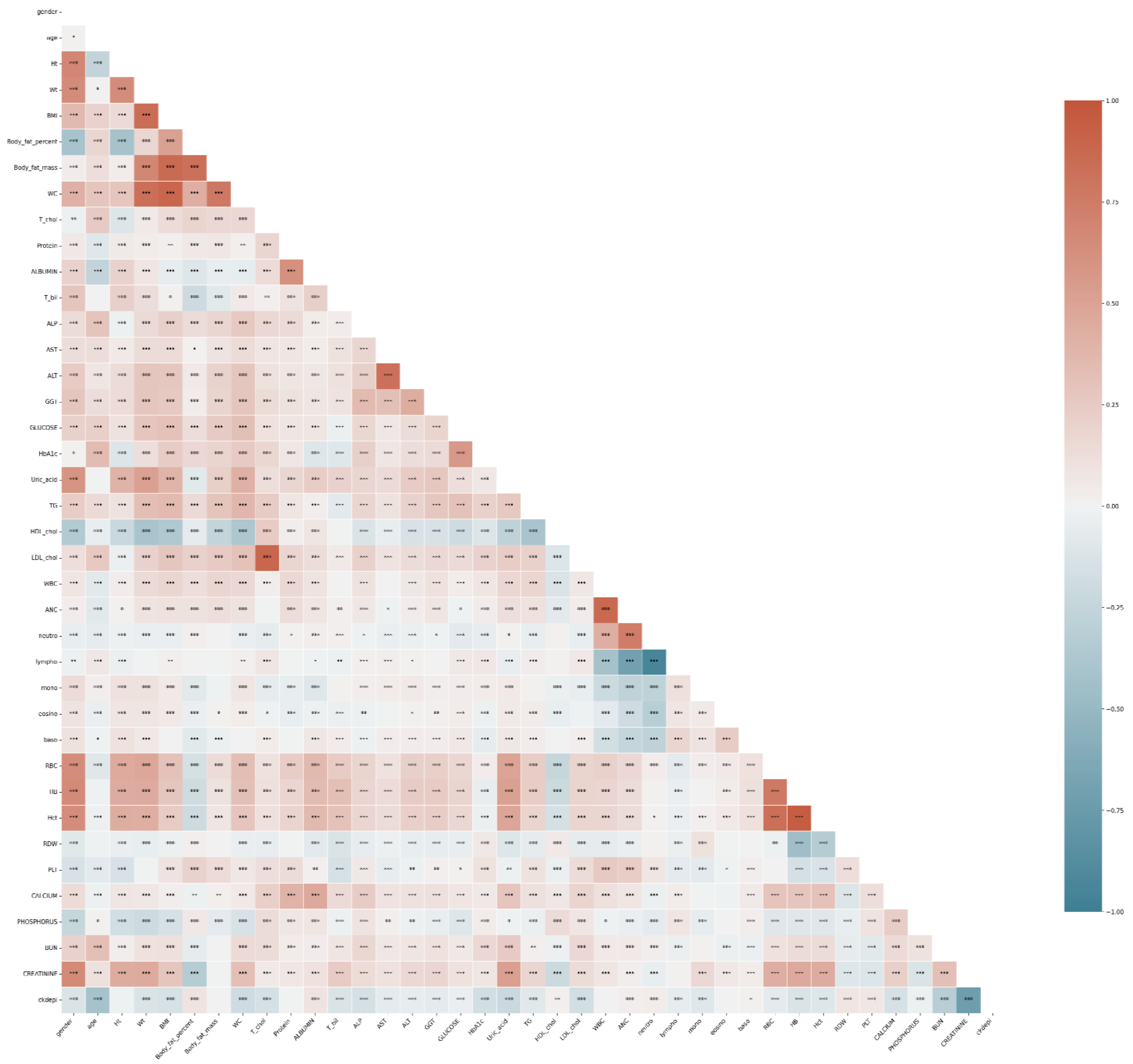

Figure S7. Correlation among the variables in GENIE cohort
